# The function and clinical implication of circular RNAs in lung cancer

**DOI:** 10.3389/fonc.2022.862602

**Published:** 2022-10-19

**Authors:** Wenjun Ren, Yixiao Yuan, Jun Peng, Luciano Mutti, Xiulin Jiang

**Affiliations:** ^1^ Department of Cardiovascular Surgery, The First People's Hospital of Yunnan Province, Kunming, Yunnan, China; ^2^ Department of Thoracic Surgery, The Second Affiliated Hospital of Kunming Medical University, Kunming, Yunnan, China; ^3^ Key Laboratory of Molecular Oncology and Epigenetics, The First Affiliated Hospital of Chongqing Medical University, Chongqing, China; ^4^ Department of Thoracic Surgery, The First People's Hospital of Yunnan Province, Kunming, Yunnan, China; ^5^ The Sbarro Institute for Cancer Research and Molecular Medicine, Center for Biotechnology, College of Science and Technology, Temple University, Philadelphia, PA, United States; ^6^ Kunming College of Life Science, University of Chinese Academy of Sciences, Beijing, China

**Keywords:** circRNA, lung cancer, clinical significance, diagnostic, mechanism

## Abstract

Lung cancer is the leading cause of cancer-related deaths worldwide. Despite the recent advent of promising new targeted therapies, lung cancer diagnostic strategies still have difficulty in identifying the disease at an early stage. Therefore, the characterizations of more sensible and specific cancer biomarkers have become an important goal for clinicians. Circular RNAs are covalently close, endogenous RNAs without 5′ end caps or 3′poly (A) tails and have been characterized by high stability, abundance, and conservation as well as display cell/tissue/developmental stage-specific expressions. Numerous studies have confirmed that circRNAs act as microRNA (miRNA) sponges, RNA-binding protein, and transcriptional regulators; some circRNAs even act as translation templates that participate in multiple pathophysiological processes. Growing evidence have confirmed that circRNAs are involved in the pathogenesis of lung cancers through the regulation of proliferation and invasion, cell cycle, autophagy, apoptosis, stemness, tumor microenvironment, and chemotherapy resistance. Moreover, circRNAs have emerged as potential biomarkers for lung cancer diagnosis and prognosis and targets for developing new treatments. In this review, we will summarize recent progresses in identifying the biogenesis, biological functions, potential mechanisms, and clinical applications of these molecules for lung cancer diagnosis, prognosis, and targeted therapy.

## Introduction

According to the cancer statistics of 2020, lung cancer is the second most frequent human tumor and the leading cause of cancer-related deaths worldwide (1). Lung cancer includes small-cell lung carcinoma (SCLC) and non-small-cell lung carcinoma (NSCLC). NSCLC accounts for approximately 85% of all cases (2) and is mostly represented by lung adenocarcinoma (ADC), lung squamous cell carcinoma (SCC), and large-cell lung carcinoma.

Despite the recent rapid advances in diagnostic tumor biomarkers and therapeutic approaches such as surgery, chemotherapy, radiotherapy, targeted therapy, or immunotherapy, the 5-year overall survival for lung cancer still remains poor (3). Therefore, finding new biomarkers and therapeutic targets for this cancer is paramount.

The initiation and progression of lung cancer is an extremely complex process that involves genetic mutations, the role of the tumor microenvironment, and the dysregulation of epigenetic pathways (4–6). Epigenetic changes in lung cancer such as histone modifications (7), DNA methylation (8), and noncoding RNAs (ncRNAs) (9) have been extensively studied. Cellular ribonucleic acids (RNAs) can be divided into coding and non-coding RNAs; these latter kind include small interfering RNAs (siRNAs), small nuclear RNAs (snRNAs), small nucleolar RNAs (snoRNAs), PIWI-interacting RNAs (piRNAs), transfer RNAs (tRNAs), ribosomal RNAs (rRNAs), microRNAs (miRNAs), long non-coding RNAs (lncRNAs), and circular RNAs (circRNAs) (10–12).

Several evidence have revealed that numerous ncRNAs are dysregulated and involved in lung cancer initiation and progression (13–15). In particular, miRNAs and lncRNAs have been shown to be involved in lung cancer progression—for example, microRNA-485-5p suppresses growth and metastasis in non-small-cell lung cancer cells by targeting IGF2BP2 (16), whereas miRNA-124 suppresses cell proliferation in NSCLC by the down-regulation of SOX8 expression (17), and lncRNA H19 promotes lung cancer proliferation and metastasis by inhibiting the miR-200a function (18). lncRNA TUC338 promotes the invasion of lung cancer by activating the MAPK pathway (19). While the role of miRNAs and lncRNAs in cancer development is currently being progressively unraveled, the functions of circRNA in the initiation and progression of lung cancers need further investigation.

As a new type of ncRNAs with distinct properties and diverse functions, unlike lncRNA and miRNA, circRNA is an endogenous RNA molecule with covalently closed loop structures without termination at 5′ caps and 3′ poly(A) tails. The first circRNA was observed in viroid (20). Next, circRNAs were discovered in the cytoplasm of eukaryotic cells by electron microscopy (21) and were earlier mainly considered to be “junk RNAs” produced by aberrant splicing events (22), until it was hypothesized as a possible function of the testis-specific circRNA, expressed by Mus spretus Sry genes (23). Recently, with the development of high-throughput RNA sequencing and bioinformatics algorithms, thousands of circRNAs in eukaryotes have been identified, including plants, fungi, protists, fish, worms, and mammals (24–26). circRNAs are composed of exonic and/or intronic sequences and are primarily generated by back-splicing, a non-canonical alternative RNA splicing event mediated by the spliceosome and regulated by a combination of cis-elements and trans-factors (27, 28). According to biogenesis from a different mechanism, circRNA can be divided into three types: exonic circRNA (ecircRNNAs) (29), intronic RNAs (ciRNAs) (30), and exon–intron circRNAs (EIcircRNA) characterized by the co-presence of both exons and introns (30).

Despite the lack of polyadenylation [poly(A)] and capping, circRNAs generally localize to the cytoplasm (29), while the exon–intron circRNAs and ciRNAs generally localize in the nucleus and promote the transcription of their parental genes *via* interaction with the U1 small nuclear ribonucleoprotein (31). circRNAs are characterized by abundance (32), stability (33), translational capacity (34), and cell specificity/tissue specificity/developmental stage specificity (29, 35). Numerous studies have demonstrated that circRNAs play a critical role in gene expression (36), act as miRNA sponge (37), interact with RNA binding protein (38), and translate to small peptides (39). Moreover, circRNAs could play paramount roles in physiological processes, including aging (40), myogenesis (41), male reproductive function (42), adipogenesis (43), innate immune response (28), synaptic function (44), insulin secretion (45), and mitochondrial ROS (mROS) output (46). On the other hand, dysregulation circRNAs are also involved in various human diseases, including neurological disorders (47), cardiovascular diseases (32), chronic inflammatory diseases (48), diabetes mellitus (49), and especially human cancers (50, 51).

Liquid biopsy is a biopsy that uses body liquids as the sample source to diagnose, predict the outcome of, or monitor the development of human diseases (52), and because of their high stability, abundant expression, and high specificity, circRNAs detected with liquid biopsy could become promising biomarkers for human diseases (53). circRNAs are involved in the pathogenesis of lung cancer through the regulation of proliferation and invasion (54), cell cycle (55), stemness of lung cancer stem cell (56), chemotherapy resistance (57), and tumor microenvironment (58, 59). Therefore, we will summarize recent progresses in identifying the biological and prognostic role of circRNAs and their potential exploitation as actionable targets in lung cancer.

## Biogenesis and properties of circRNAs

### The classification and biogenesis of circRNA

circRNAs are generated by RNA polymerase II from pre-mRNA (60). These circRNAs are distinct from their linear RNA counterparts because they lack the 5′–3′ ends and poly-adenylated tail due to their closed covalent structure, which usually decides the fate of many RNA transcripts (29). According to the generation mechanism, the circRNAs can be divided into exonic circRNAs (ecircRNAs) originated from one or more exonic sequences (61) and intronic circRNAs (ciRNAs) originated from intronic sequences (30), while the exon–intron circRNAs (EIciRNAs) can be produced from intron-containing exons (62). Exonic circRNAs are cytoplasmic and result from pre-mRNA splicing where the 3′ splice donor attaches to the 5′ splice acceptor forming an exonic circRNA (61). Exon–intron circRNAs are predominantly nuclear and composed of introns and exons that interact with U1 snRNP and promote the transcription of their parental genes (31). Intronic RNA (ciRNA) formation depends on the 7-nt GU-rich element near the 5′ splice site and an 11-nt C-rich element close to the branchpoint site (30).

At the moment, there are several hypotheses to explain circRNA biogenesis, including (1) intron-pairing-driven circularization (2), RNA-binding protein (RBP)-driven circularization (3), exon skipping, and (4) intron lariat circularization (63).

The intron-pairing-driven circularization model suggests that introns flanking the exon/exons of a pre-mRNA have a structure capable of joining each other. The flanking introns approach each other, creating a secondary conformation that makes the splice sites possibly carry on back-splicing and generate exonic circRNA. Adenosine deaminase 1 acting on RNA (ADAR1) is involved in the intron pairing process of circRNA formation (64).

In the RBP-driven circularization model, RBPs bind to pre-mRNAs to connect the flanking introns. This process is induced by protein dimerization, which forms an RNA loop. Muscle blind-like splicing regulator 1 (MBNL1) protein is the most frequent RBP responsible for circRNA biogenesis (65). In the exon skipping model, one or multiple exons of the mature mRNA will be missing, whereas the lariat-driven circularization hypothesis is based on the binding of adjacent exons, leading to the formation of linear mRNA and exon–intron or multiple-exon circRNA transcript with lariat structure. Finally, the fourth proposed mechanism is the intron lariats, which can form ciRNAs (66).

### Role of cis-elements and trans-factors in circular RNA formation

**Figure 1:**
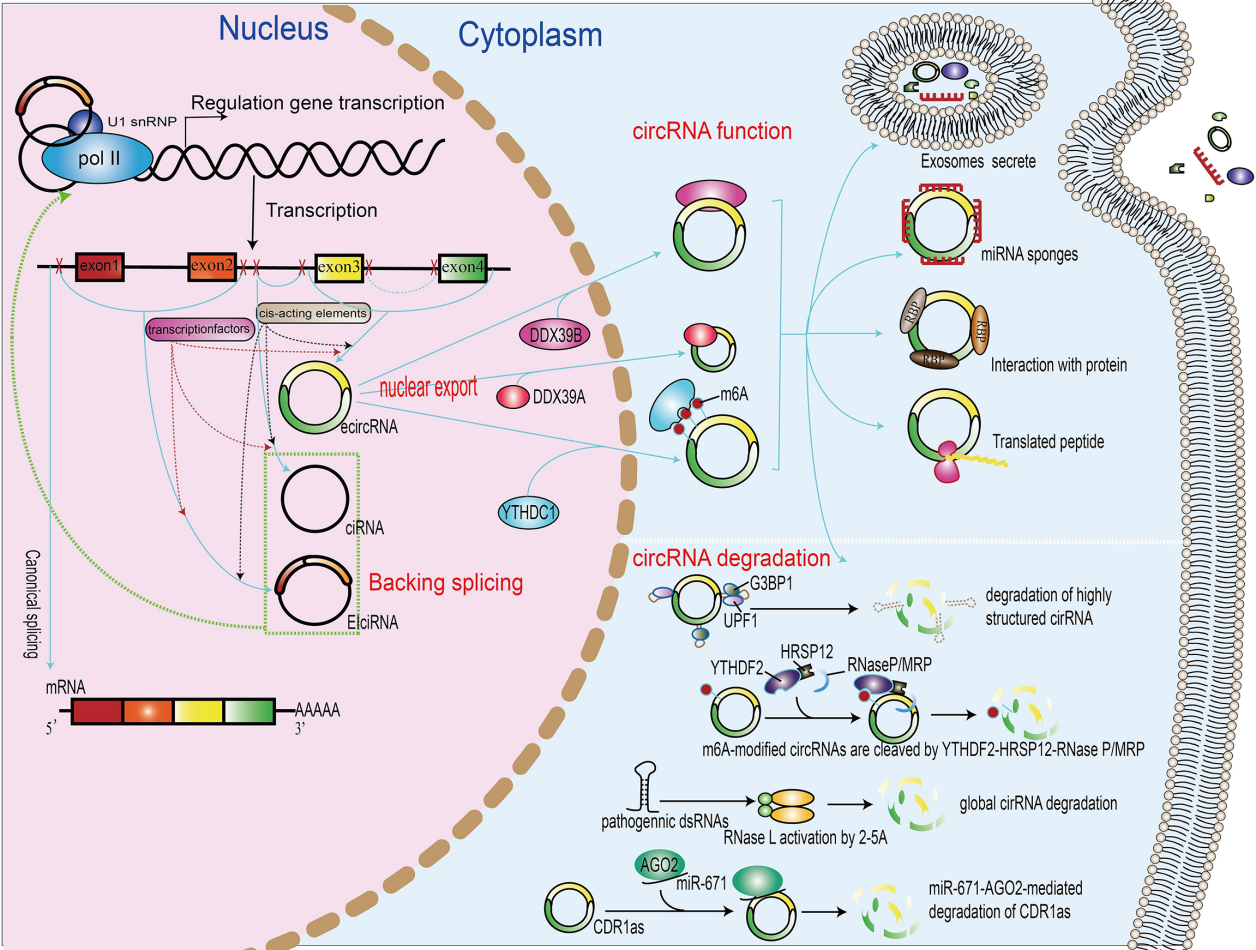
Biogenesis, nuclear export, functions, and degradation of circRNAs. In the nucleus of eukaryotic cells, DNA is transcribed to form precursor mRNA (pre-mRNA), which contains coding exons and introns. Differently from linear mRNAs, which are formed regulated by cis-acting elements and transcription factors, according to the generation mechanism, the circRNAs can be divided into exonic–intronic circRNAs (ecircRNAs), ciRNAs, and circRNAs (EIciRNAs). The proteins UAP56, URH49, and YTHDC1 can promote the nuclear export of circRNAs. The functions of circRNAs include the regulation of gene transcription or splicing, acting as miRNA sponge, binding with RNA-binding protein, translation into peptide or protein, and packing in exosomes. The degradation of circRNA includes miRNA-directed circRNA decay, m6A-mediated circRNA decay, RNase L-mediated circRNA decay, and overall structure-mediated circRNA decay.

Mounting evidence have shown that back-splicing requires spliceosomal machinery and that the regulation of circRNA formation depends on both cis-regulatory elements and trans-acting factors. In this review, we shall focus on how cis-regulatory elements and trans-acting factors influence the biogenesis of circRNA (**Figure 1**).

#### Cis elements regulate the formation of circRNAs

Back-splicing often requires regulatory elements residing in introns flanking circularized exons. Flanking intronic complementary sequences (ICSs) can promote exon circularization and then give rise to the formation of circRNAs (27). Most circRNAs in mammals are produced from internal exons with long flanking introns usually containing ICSs. RNA pairing formed across introns that flank back-spliced exons is expected to bring the distal single strand into close proximity to facilitate circRNA biogenesis (61). Consistently, the elimination of RNA pairs significantly reduces and, sometimes, even removes circRNA production, as revealed by mutagenesis analysis in circRNA expression vectors (67, 68)—for example, in cultured mouse cells, a previous study showed that circSry, derived from Sry gene, is flanked by many intronic complementary sequences, and the presence of long inverted repeats (IR) flanking of Sry gene results in the formation of the Sry circular transcript (69). Furthermore, the fusion-circRNAs (f-circRNAs) that originated from aberrant chromosomal translocations in cancers also supported the view that intronic RNA pairing across circle-forming exons is critical for enhanced circRNA formation (70). F-circM9 was identified to be produced by the MLL/AF9 fusion gene that contains the MLL gene exon 1–8 and AF9 gene exon 6–11. After translocation, the trans intronic sequences of the MLL and AF9 genes are juxtaposed in cis, which subsequently facilitates circRNA generation by forming newly paired ICSs flanking the translocation breakpoint (70). ICSs even have 23 nucleotides flanking intronic regions and also promote the formation of natural circRNA in platelets (27). It has been shown that Alu elements are abundant and constitute 11% of the reference human genome, and nearly half of them is located in human introns (71). A recent study demonstrated that complementary inverted-repeat Alu elements drive the RNA pairs, which enhances the exon circularization efficiency and leads to multiple circular RNA transcripts produced from a single gene (27). RNA pairing formed across flanking introns generally promotes circRNA formation (72). Furthermore, RNA pairing within individual introns generally facilitates canonical splicing in linear RNA, competing with RNA pairing across flanking introns and leading to reduced circRNA formation from the same gene locus (36). These recent findings together reveal that cis elements play a key role in circRNA biogenesis.

#### Trans-acting factor regulate the formation of circRNAs

Previous studies have demonstrated that RNA-binding proteins are also involved in spliceosome function *via* the modulation of alternative splicing which control the information of circRNAs. In Drosophila cells, using RNAi depletion or the pharmacological inhibition of spliceosome-related gene expression inhibits canonical pre-mRNA processing and increases the output of circular RNAs (73). Another study likewise shows that the pharmacological inhibition of the spliceosome promotes the biogenesis of circRNAs in mouse brain (74). Therefore, one can assume that spliceosome regulates the activity of back-splicing and canonical splicing and then affects the biogenesis of circRNAs and mRNA. The abundance of core spliceosomal factors promotes the biogenesis of circRNAs and reduces the product of linear mRNA. The RNA-binding proteins regulate the back-splicing process through an interaction with ICSs, whereas the double-stranded RNA-binding domains in RBPs bind to ICSs and stabilize the transiently formed intronic RNA pair flanking—for example, the NF90/NF110 encoded by interleukin enhancer binding factor 3 gene, which includes a double-stranded RNA-binding domain, *via* binding to intronic inverted-repeat Alu elements, promotes circRNA production (28). The immune factors NF90 and/or NF110, each containing two dsRNA-binding domains (dsRBDs), promote circRNA formation by direct binding to IRAlus formed in nascent pre-mRNA. In the endogenous NF90 knockdown condition, the re-introduction of wild-type NF90, but not NF90 mutants with dsRBD truncations, rescued circRNA expression, further supporting that both dsRBDs of NF90 are required for circRNA production (28). However, dsRBPs can also inhibit circRNA formation by destabilizing the RNA pairing—for example, it was reported that the depletion of DHX9 from nuclear RNA helicase, known to bind to inverted-repeat Alu elements, thus regulating the biogenesis of circRNAs, leads to the double-stranded RNA accumulation defects and increased the circular RNA production (75). ADAR1, a double-strand RNA-editing enzyme, edits A-to-I of inverted-repeat Alu elements flanking circRNA-forming exons and significantly increases circRNA expression (24). RNA-binding proteins without a dsRBD domain also participate in the regulation of circRNA levels by direct binding to specific RNA motifs—for instance, Quaking (QKI), which is an alternative splicing factor, promotes the interaction between QKI motifs and introns flanking circRNA-forming exons, thus leading to the biogenesis of hundreds of circRNAs with a paramount role in human epithelial–mesenchymal transition (EMT) (76). Other splicing factors have been found to regulate back-splicing in different biological settings: RBM20 promotes the biogenesis of circRNAs by regulating the exclusion of specific exons (77) and heterogeneous nuclear ribonucleoprotein L (HNRNPL) regulates back-splicing and promotes the biogenesis of circRNAs and tumor growth in prostate cancer (76). Splicing factor-regulated back-splicing events also occur in other species. In Drosophila melanogaster, the splicing factor Muscle blind (Mbl) regulates circRNA production from its own pre-mRNA by binding to multiple Mbl-binding sites in introns flanking the circularized exon in Mbl pre-mRNA (36). These recent findings together revealed that circRNA production is highly dependent on biological background and tightly regulated in cells using different cis elements and trans factors that are back-splicing specific.

### The properties of circRNAs

circRNAs have the characteristics of abundance (32), specificity (25), stability (33), and translational capacity (34). It has also been shown that circRNAs usually have higher expression levels in low-proliferating cells, and developing tissues such as the heart, lung, and brain usually show higher levels of circRNA (78). These evidence show that the expression patterns of circRNAs are tissue and development specific. While some studies found that the expression of circRNAs was not correlated with their host mRNAs, only in some special circumstances were the expression levels of circRNAs higher than those of their host mRNAs (79). Most of the circular RNAs are cytoplasmic due to the lack of 5′ caps and 3′ poly-A tails that confer resistance to ribonuclease R (RNase R)-dependent degradation (33). The half-life of circRNAs far exceeds that of their linear RNA counterparts (48 and 10 h, respectively (61)). These characteristics make circRNAs ideal biomarkers of cancer.

Numerous results have shown that the expression of circRNAs exhibit specificity to certain tissues and developmental stage—for example, one study found that circRNAs were extraordinarily enriched in the mammalian brain compared to other tissues (80).

Although circRNAs belong to non-coding RNAs, recent studies unraveled that circRNAs can be translated into proteins or peptides—for example, circ-FBXW7 encodes a novel 21-kDa protein, called FBXW7-185aa, and the depletion of FBXW7-185aa promotes malignant phenotypes in glioma (81).

### The nuclear export of circular RNAs

In spite of the fact that most circRNAs are primarily cytoplasmic while circRNAs containing retained introns are nuclear (31), some circRNAs are formed in the nucleus and then transported to the cytoplasm.

Their elaborated control of transport is essential for circular RNAs to exert their functions properly in eukaryotic cells. A recent study has demonstrated that m6A modification and other proteins modulate the circRNA export and that knock-down of the DExH/D-box helicase Hel25E leads to the nuclear accumulation of circRNAs longer than 800 nucleotides. Moreover, the depletion of UAP56 or URH49 results in long and short circRNA enrichment in the nucleus, respectively (82). These evidence indicate that the lengths of mature circRNAs may be a decisive factor for their nuclear export. In addition, a more recent study suggest that N6-methyladenosine RNA modification plays a significant role in the regulation of the nuclear export YTHDC1, a circRNA m6A reader that mediates the export of methylated mRNA from the nucleus to the cytoplasm as demonstrated by the depletion of YTHDC1 that leads to the accumulation of m6A-containing transcripts in the nucleus. Another study demonstrated the interaction of YTHDC1 with the splicing factor and nuclear export adaptor protein SRSF3, leading to the increased nuclear export of m6A-containing transcripts (83).

Very similarly, in colorectal carcinoma patients, Chen et al. recently identified the pro-metastatic effect of the upregulation of the m6A-modified circRNA circNSUN2 that enhances the stability of high-mobility group AT-hook protein 2 mRNA. Furthermore, the m6A modification increases the export of circNSUN2 to the cytoplasm (84) (**Figure 1**).

To conclude, it can be assumed that the mechanisms for extracellular transportation are not yet fully understood, and further studies on this function are certainly warranted.

### The degradation of circular RNAs

The circular structure confers resistance of circRNAs to degradation by RNA decay nuclease and other proteins. Therefore, circRNAs have much higher stability than their cognate linear transcripts (85). Nevertheless, under stress conditions, circRNAs can also be degraded through different mechanisms. Thomas Hansen et al. revealed that miRNAs directly bind circRNAs and promote their degradation process. miR-671 directly cleaves a circular antisense transcript of the CDR1 locus in an Ago2-slicer-dependent manner, whereas the miR-671 target site of the circRNA CDR1as/ciRS-7 recruits miR-671-loading Ago2 to CDR1as/ciRS-7, causing endonucleolytic cleavage by Ago2 and subsequent exonucleolytic RNA degradation (86). In addition, many endonucleases participate in the circRNA degradation process. Under viral infection, double-stranded RNA (dsRNA) activates the RNase L that, in turn, promotes the degradation of circRNAs (87). Fischer et al. have revealed that, during this novel structure-mediated circRNA decay, UPF1 interacts with G3BP1 and accelerates the structure-mediated circRNA decay degradation (88). Interestingly, N6-methyladenosine RNA modifications also affect the degradation of circRNAs; m6A-containing circRNAs use m6A as a recruiter of the m6A reader protein YTHDF2 and adaptor protein HRSP12. Eventually, HRSP12 directly binds to a GGUUC motif on circRNAs and serves as a bridge to bring YTHDF2 and endoribonuclease RNase P/MRP together, thus enabling RNase P/MRP to initiate circRNA degradation (89) (**Figure 1**).

## The roles of circRNAs in physiological conditions

Previous studies have demonstrated that circRNAs contribute to gene regulation through a variety of actions, including regulating transcription, sponging microRNAs, interacting with RNA-binding proteins, and protein translation. The potential functions of circRNAs have been extensively studied (**Figure 1**). A number of studies have begun to reveal that at least some circRNAs could play crucial roles in physiological conditions by distinct modes of action at the molecular level.

### circRNAs regulate transcription and splicing

ciRNAs and EIciRNAs are the products of processed intron lariats and back-splicing with retained introns, respectively. Both of them are mostly nuclear and regulate gene expression at the transcriptional and post-transcriptional levels (31). EIciRNAs interact with U1 small nuclear ribonucleoproteins (snRNP) and RNA PoII in the promoter region of the parental gene to enhance gene expression in cis—for instance, the blockage of circEIF3J and circPAIP2 reduces the transcriptional level of host genes (31). As expected, the processing of circRNAs affects the alternative splicing of their linear cognates and competes with pre-mRNA splicing, suggesting a negative relationship between circRNA and their linear isoforms (36)—for example, circMbl derived from the circularization of the second exon of the MBL competes with linear MBL mRNA to maintain the balance between canonical splicing and circRNA production (36). However, in addition to exon skipping, whether there are additional regulators that affect exon circularization remains to be explored. Recently, a study showed that circSEP3, derived from exon 6 of the SEPALLATA3 gene, concurs to form a RNA–DNA complex that participates in the transcription pausing and alternatively splicing process of the SEP3 gene (31). The above-mentioned research shows that some localized nuclear circRNAs can modulate gene transcription and splicing *via* different mechanisms

### circRNAs act as miRNA sponges

A scenario in which competitive endogenous RNA (ceRNA) transcripts with shared microRNA (miRNA) binding sites compete for the post-transcriptional control expression of mRNA is hypothesized (90). In the case of cytoplasmic circRNAs, it acts as miRNA sponge and stabilizes mRNA stability, preventing miRNA-mediated degradation. There are many evidences showing that circRNAs act as miRNA sponges: as a sponge for miR-7, ciRS-7 contains more than 70 selectively conserved miR-7 target sites, is highly and widely connected with AGO proteins, strongly suppresses miR-7 activity, and increases the expression of miR-7 targets (37). circRNA Sry, the testis-specific circRNA in sex-determining region Y (Sry), is a potent miRNA 138 sponge that contains 16 putative target sites for miR-138 and directly binds miRNA 138 to participate in the development of mouse testis (37). Similarly, a recent study indicated that circHIPK3, derived from Exon2 of the HIPK3 gene, contains many potential binding sites of miR-124 and acts as a sponge-inhibiting miR-124, an activity with subsequent pivotal biological effects in human cells (91). It has been shown that circZNF91, sponge for miR-23b-3p, modulates human epidermal stem cell differentiation (92), whereas circBIRC6 directly binds miR-34a and miR-145 and modulates the pluripotency and differentiation of human embryonic stem cells (93). These evidence suggest that circRNAs play a crucial role in controlling the stability and quantity of miRNAs.

### circRNAs interact with RNA-binding proteins

Although circRNAs mostly act as miRNA sponges, many research studies have been carried out to explore the functional relevance of circRNA–protein interactions. The most well-known proteins interacting with RNA molecules are the RNA-binding proteins (RBPs). RBPs contain a large class of over 2,000 proteins that interact with transcripts and play an important role in RNA metabolic processes (94). Previous evidence have shown that circRNAs interact with RBPs to form specific circRNA–protein complexes and affect protein localization and function. Exon–intron circRNAs interact with U1 snRNP and regulate the expression of their related parental genes (31). Circular RNA circ-Foxo3 is mostly cytoplasmic, where it interacts with the anti-senescent protein ID-1, the transcription factor E2F1, FAK, and HIF1α, resulting in increased cellular senescence (95). circ-Foxo3 interacts with cell cycle protein cyclin-dependent kinase 2 and cyclin-dependent kinase inhibitor 1 and leads to the formation of a ternary complex, thus promoting the cell cycle progression of non-cancer cells (96). Although circ-Foxo3 is minimally expressed in tumor specimens, the expression of circ-Foxo3 gradually increases during cancer cell apoptosis. Other studies demonstrate that circ-Foxo3 and MDM2/p53 form the complex circ-Foxo3/MDM2/p53 that promotes MDM2-induced p53 ubiquitination and subsequent degradation. circ-Foxo3 plays an important regulatory role in cell apoptosis because it prevents MDM2 from inducing Foxo3 ubiquitination and degradation, resulting in elevated levels of Foxo3 protein (97). It has also been reported that circACC1 interacts with AMPK and elevates the stability of AMPK holoenzyme, promotes the enzymatic activity of the AMPK holoenzyme by forming a ternary complex with the regulatory β and γ subunits of AMPK holoenzyme, and plays a critical role in both fatty acid β-oxidation and glycolysis (98). Abdelmohsen K and colleagues provided evidence that CircPABPN1 prevents HuR binding to PABPN1 mRNA that results in the suppression of PABPN1 translation (99). Another typical example is circ-AMOTL1, which directly binds to PDK1 and AKT1, leading to AKT1 phosphorylation and nuclear translocation, and plays a cardio-protective role in cardiovascular disease (100). Furthermore, in GBM, circSMARCA5 acts as sponge for SRSF1 as well as participates in the process of VEGFA mRNA splicing regulation and angiogenesis (101). In summary, these evidence indicated that circRNAs interact with RNA-binding proteins and play a significant role in protein function regulation.

CircANRI binds pescadillo homologue 1 (PES1) and is an essential 60S-preribosomal assembly factor that impairs exonuclease-mediated pre-rRNA processing and ribosome biogenesis in vascular smooth muscle cells and macrophages (48). CircHECTD1 promotes fibroblast proliferation and migration *via* interaction with HECTD1/ZC3H12A (102). CircZKSCAN1 competes with the binding between FMRP and β-catenin-binding protein cell cycle and apoptosis regulator 1 (CCAR1) mRNA, with subsequent inhibition of WNT signaling and HCC stemness (103). CircARSP9 increases the susceptibility of HCC cells to NK cell cytotoxicity in HCC cells by the upregulation of UL16-binding protein (ULBP1) expression (104). AR suppresses CircARSP91 expression by upregulating ADAR1, while CircARSP91 plays an inhibitory role in HCC tumor growth (105). Circular RNA FECR1 controls breast cancer tumor growth by the recruitment of TET1 to FLI1 promoter, determining the over-expression of FLI1 (106). circRNA−MTO1 modulates the Eg5 protein expression, suppresses cell viability, promotes monastrol-induced cell cytotoxicity, and reverses monastrol resistance (107) via tumor necrosis factor receptor-associated factor 4 (TRAF4). Circ-Dnmt1 interacts with both p53 and AUF1, promotes the nuclear translocation of both proteins, and plays an oncogenic role in breast cancer cell autophagy (108). CircECE1 was reported to interact with c-Myc to prevent speckle-type POZ-mediated c-Myc ubiquitination and degradation and exert the proliferation and metastatic capability of osteosarcoma cells (109). circRNAs interact with proteins to influence their cellular functions, thereby regulating gene transcription and inhibiting cell cycle progression but promoting cardiac senescence, apoptosis, and cell proliferation.

### circRNAs can be translated

Due to lack of 5–3′ polarity, polyadenylated tails, and internal ribosome entry sites (IRES), circRNAs were initially defined as a noncoding RNA. Notwithstanding, a recent study found that, under certain conditions, circRNAs possess translational ability and can code functional peptides—for example, Circ-ZNF609 contains an open reading frame span that can be translated into a protein that controls myoblast proliferation (39). Moreover, m6A modification regulates circRNA translation and METTL3 and YTHDC1 regulate back-splicing, while the knockdown of METTL3 downregulates Circ-ZNF609.

Another study shows that YTHDF3 and eIF4G2 recognize the m6A modifications in Circ-ZNF609 and modulate its translation (110). It was suggested that circular RNA derived from the long intergenic non-protein-coding RNA p53-induced transcript (LINC-PINT) can code an 87-amino-acid peptide. This peptide directly interacts with PAF1c, inhibits the transcriptional elongation of multiple oncogenes, and plays a suppressive role in glioblastoma cells (111). Circ-FBXW7, highly expressed in normal human brain, has been reported to encode a novel 21-kDa protein, named FBXW7-185aa, that reduces the half-life of c-Myc by antagonizing USP28-induced c-Myc stabilization, thus acting as tumor suppressor in glioblastoma cells (34). In addition, circ-SHPRH contains an open reading frame.

The IRES translate a functional protein named SHPRH-146aa and play an additional inhibitory effect in GBM, protecting the full-length SHPRH from ubiquitination (112). Similarly, circβ-catenin is highly expressed in liver cancer tissues where it promotes liver cancer cell growth. Another study shows that Circβ-catenin is predominantly localized in the cytoplasm and encodes a novel 370-amino-acid β-catenin isoform *via* a linear β-catenin mRNA. This β-catenin isoform increases the stability of full-length β-catenin by antagonizing GSK3β-induced β-catenin phosphorylation and degradation and eventually results in the activation of the Wnt pathway (113). Circ PPP1R12A contains an open reading frame encoding the functional protein circPPP1R12A-73aa that promotes the growth and metastasis of colon cancer *via* activating the Hippo-YAP signaling pathway (114).

We named AKT3-174aa as circ AKT3 encoding a 174-amino-acid novel protein that competes with phosphorylated PDK1, reduces AKT-thr308 phosphorylation, and plays an inhibitory role in the tumorigenicity of GBM cells (115).

CircE7 is derived from human papillomaviruses and, *via* its interactionwith polyribosomes, is translated into the E7 oncoprotein. E7 oncoprotein depletion significantly suppresses cancer cell growth and tumor xenografts (116). CircLgr4 is highly expressed in colorectal tumors and colorectal cancer stem cells. The knockdown of circLgr4 inhibits colorectal cancer stem cell (CSC) self-renewal, colorectal tumorigenesis, and invasion. CircLgr4 encodes the circLgr4-peptide, which interacts with LGR4 to activate the LGR4-Wnt signaling pathway that, in turn, drives the self-renewal of colorectal CSCs (117). In colon cancer cell lines and tissue, circ FNDC3B is mostly localized in the cytoplasm, and its over-expression inhibits the proliferation, invasion, and migration of colon cancer cells. CircFNDC3B also encodes a novel protein circFNDC3B-218aa that suppresses colon cancer progression (118). These evidence pave the avenue to future studies on the translational function of circRNA.

### Other physiological functions

A growing body of evidence show that circRNAs play a critical role in the physiological processes, including exosomes secretion (119), aging (40), myogenesis (41), male reproductive function (42), adipogenesis (43), innate immune response (28), synaptic function (44), insulin secretion (45), and mROS output (46). Exosomes are extracellular membranous micro-vesicles with a diameter of 40–160 nm and secreted by various cell types (120). Exosomes derived from host cells can be taken up and exert biological effects both on adjacent and distant cells (121). Exosomes have been implicated in the occurrence and development of many diseases, including cancer (122). Exosomes may include biological molecules such as lipids, proteins, DNA, and non-coding RNAs (ncRNAs), and their content and biological effects depend on the host cells.

ncRNA-containing exosomes play a role in disease progression, including cancer (123). A recent study by Zhang N et al. reported that circSATB2 is highly expressed in NSCLC cancer exosomes and promotes the proliferation, migration, and invasion of NLCSC cells. SATB2-containing exosome can be taken up by NLCSC cells to promote cell–cell communication, progression of NSCLC cells, proliferation of normal bronchial epithelial cells, and lymphatic spreading. The receiver operating characteristic (ROC) analysis curve of exosomial circSATB2 shows an area under the ROC curve (AUC) value of 0.660 and 0.797 in serum from patients with lung cancer and metastatic lung cancer patients, respectively. These results indicate that exosomial circSATB2 could act as a blood detection index for the diagnosis of lung cancer and lung cancer metastasis with high sensitivity and specificity (124). Similarly, it has also been reported that circRNA 002178 exosomes from the plasma of LUAC patients were highly expressed in LUAC tissues and LUAC cancer cells. Functional studies have demonstrated that circRNA-002178 enhances PDL1 and induces T cell exhaustion *via* sponging miR-34 in cancer cells. Interestingly, circRNA-002178 could be exploited as a novel diagnosis biomarker for lung cancer because the AUCs have been demonstrated to be higher (0.9956) in the exosomes derived from cancer cells than in those derived from normal human bronchial epithelial cells (125).

Other evidence indicate that circRNAs play a crucial role in the mitochondria, including non-alcoholic steatohepatitis pathogenesis. The steatohepatitis-associated circRNA ATP5B Regulator (SCAR) is primarily localized in the mitochondria and alleviates meta-inflammation by reducing the mROS output. Mechanistic analyses have demonstrated that circRNA SCAR binds to ATP5B and inhibits mPTP by blocking the CypD–mPTP interaction with subsequent reduction of both mROS generation and fibroblast activation (46). Therefore, circRNA SCAR is a potential therapeutic target for nonalcoholic steatohepatitis. CircSamd4 is upregulated during the differentiation of mouse C2C12 myoblasts into myotube, whereas the overexpression of circSamd4 interferes with the binding of PUR proteins to the Mhc promoter and promotes myogenesis (41). CircArhgap5-2 promotes adipogenesis through maintaining the global adipocyte transcriptional program involved in lipid biosynthesis and metabolism (43). Flies missing circular Boule (circBoule) RNAs have decreased male fertility, under heat stress conditions, and the knockdown of circBoule decreases the fertilization capacity. Moreover, circBoule RNAs inhibits\ the spermatogenesis process by interacting with heat shock proteins (HSPs) and promoting their ubiquitination (42). CircSfl is upregulated in the brain and muscle, and its overexpression significantly extends the lifespan of fruit flies, whereas mechanistic analyses have demonstrated that circSfl is translated into a protein sharing some functions with the full-length Sfl protein encoded by the host gene and extending the lifespan of cells (39). On the whole, these studies demonstrate that circRNAs play an important role in physiological processes.

## The roles of circRNAs in lung cancer pathological conditions

**Figure 2:**
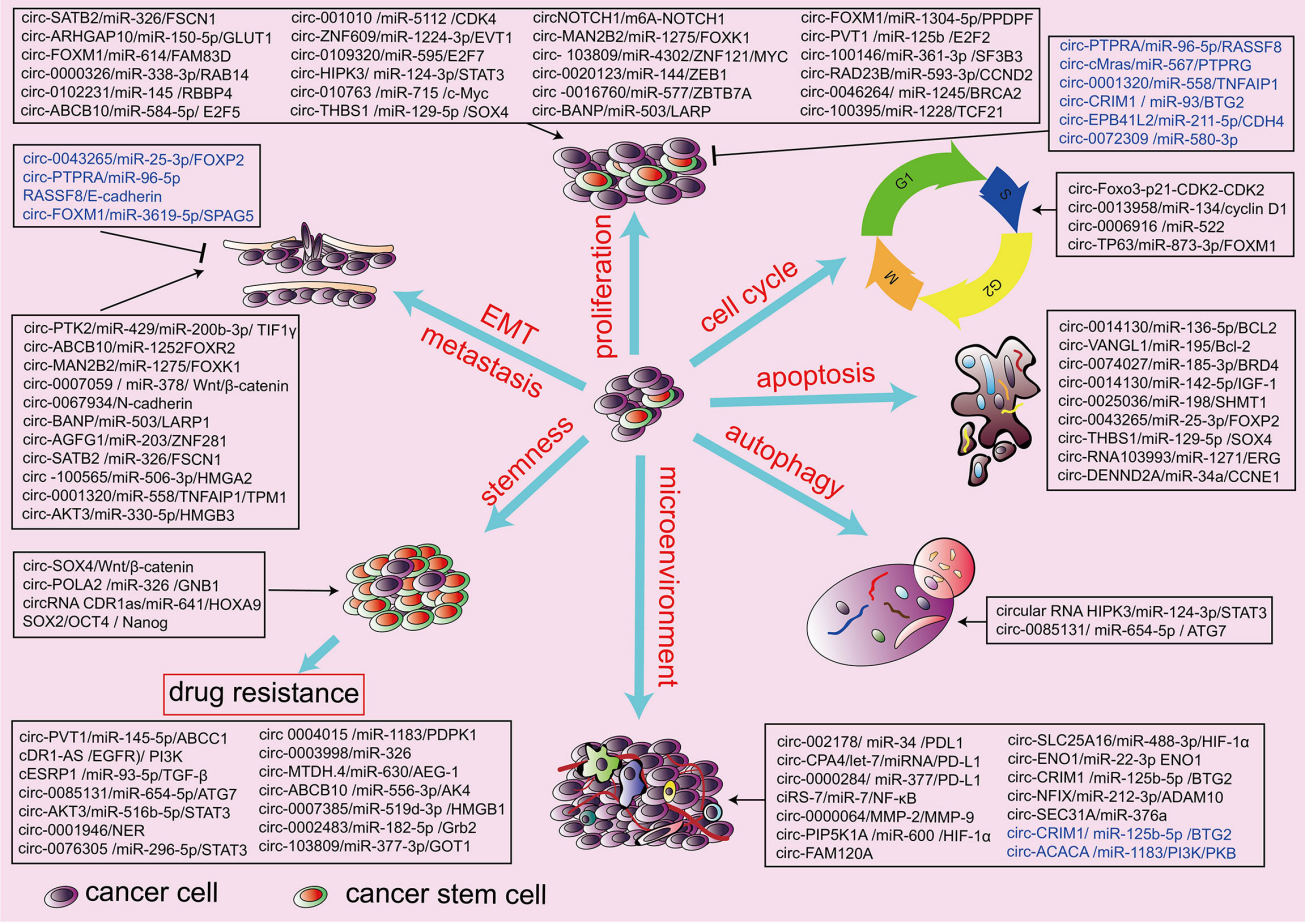
Biological function of circRNAs in the hallmarks of lung cancers. circRNAs are involved in the pathogenesis of lung cancers through the regulation of proliferation and invasion, cell cycle, cell autophagy, cell apoptosis, stemness, chemotherapy resistance, and tumor microenvironment. Black indicates an oncogenic role, and blue indicates a tumor-suppressive role.

Numerous circRNAs have been found to be dysregulated in lung cancer tissues, playing oncogenic or tumor-suppressor roles. Extensive evidence has demonstrated that there are many differentially expressed circRNAs in the tissues and plasma of lung cancer patients. Chen et al. analyzed the circular RNA expression by high-throughput sequencing of plasmatic exosomes from patients with early-stage lung adenocarcinoma and detected 182 differentially expressed exosomal circRNAs, which included 105 upregulated and 78 downregulated compared with the controls (126). Using next-generation sequencing, Zhang et al. found that 35 circRNAs were aberrantly expressed in small-cell lung cancer tissue. Among these, five circRNAs were significantly upregulated, and 30 circRNAs were significantly downregulated (127). The roles of dysregulated circRNAs in lung cancer include proliferation, migration, invasion, apoptosis, cell cycle, stemness of lung cancer stem cell, chemotherapy resistance, tumor metabolism, tumor microenvironment (TME), and immune evasion of lung cancer cells. Here we discuss biological activities and pathogenic mechanisms of cirRNAs in lung cancer (**Figure 2**).

### circRNAs regulate the proliferation and cell cycle of lung cancer cells

As the most important hallmarks of lung cancer, the proliferation of tumor cells accounts for over 50% of the cases (128). There are many evidence that circRNAs promote carcinogenesis and the progression of lung cancer, inducing cell cycle progression and proliferation through different mechanisms. Previous studies have demonstrated that endogenous F-circEA, which is derived from the EML4-ALK fusion gene, elevates the expression of F-circEA and promotes the migration and invasion ability of lung cells. F-circEA could be a novel liquid biopsy biomarker for the diagnosis of lung cancer because it is detectable in the plasma of patients with EML4-ALK translocation (129). In addition, circSATB2 is highly expressed in tumor cells and tissues and detectable in serum exosomes from patients with NSCLC. It acts as a sponge for miR-326, resulting in the upregulation of the actin-bundling protein 1 (FSCN1) expression and promoting the proliferation, migration, and invasion of NSCLC cells (124). It has been shown that circBIRC6 is upregulated in primary human NSCLC tissues and cells and, when knocked down, inhibits growth, proliferation, migration, and invasion of these tumor cells by sponging the tumor suppressor miR-145 (130). Circ NT5E promotes human NSCLC cell progression by sponging miR-134 (131), whereas circ-ARHGAP10 promotes human NSCLC cell progression *via* the miR-150-5p/GLUT1 axis (132). Circ-FOXM1 promotes the proliferation of NSCLC cells acting as sponge for miR-614, thus upregulating the expression of FAM83D (133). The high expression of Circ-0000326 in LUAC is correlated with tumor size, regional lymph node status, and differentiation. Mechanistic studies show that circ-0000326 acts as a miR-338-3p sponge and upregulates the expression of the downstream target RAB14, promoting the proliferation, migration, and apoptosis of LUAC cells (134). Circ PTPRA suppresses EMT and the metastasis of NSCLC cell lines by sponging miR-96-5p and upregulation of the downstream tumor suppressor RASSF8 (135). It has been shown that circ-0102231 is mainly localized to the cytoplasm, significantly upregulating the expression of RBBP4 (136) and promoting NSCLC cell proliferation and invasion by sponging miR-145. Circular RNA cMras inhibits LUAC progression *via* modulation HTTof the miR-567/PTPRG regulatory pathway (137). Circ-100565 modulates the expression of HMGA2 by sponging its regulatory Mir (miR-506-3p) (138). Reduction of circ-100565 significantly suppresses *in vitro* proliferation, migration, and invasion and the *in vivo* tumor growth of NSCLC cells, whereas in patients with NSCLC high levels of circ-100565 are associated with poor overall survival.

Circ-ABCB10 promotes NSCLC progression *via* modulating the miR-584-5p/E2F5 regulatory pathway (139). Cytoplasmic circ-0001320 is downregulated in lung cancer cells and inhibits the growth and invasion of lung cancer cells through the miR-558/TNFAIP1 and TPM1 pathways (126). circRNA-001010 is highly expressed in NSCLC patients and acts as a molecular sponge of miR-5112, leading to the increased expression of the oncogene CDK4 (127) with subsequent proliferation, migration, and invasion of NSCLC cells.

Knockdown of circ-0087862 significantly reduces NSCLC cell viability, migration, and invasion and enhances apoptosis, and a high expression of circ-0087862 is related with poor clinical outcome in NSCLC patients. It is hypothesized that circ-0087862 increases the expression of RAB3D by sponging miR-1253 (140). A decreased expression of Circ-CRIM1 in LUAC cancer is significantly correlated with lymphatic metastasis and more advanced TNM stage and is an independent negative biomarker for the overall survival of patients with LUAC. CircCRIM1 suppresses the invasion and metastasis of LUAC through sponging miR-93 and miR-182 and increases the expression of leukemia inhibitory factor receptor, a well-known tumor suppressor (141). circ-RNA EPB41L2 plays an inhibitory role in LUAC through regulating the miR-211-5p/CDH4 axis (142). Circ-ZNF609 is significantly upregulated in LUAC tissues and cell lines and promotes cell proliferation of LUAC cells through sponge miR-1224-3p to promote EVT1 expression (143). Circ-0072309 is lowly expressed in NSCLC tissues and cell lines and plays an inhibitory role in LUAC through blocking the expression of miR-580-3p (144). Overexpression of circ-11780 inhibits the proliferation, migration, and invasion of NSCLC cells *in vitro* and tumor growth *in vivo via* the miR-544a overexpression and reduced the protein concentration of F-Box and WD repeat domain containing 7 (FBXW7) (145).

Circ-0072088 acts as sponge for miR-377-5p, leading to the upregulation of NOVA2, and promotes the proliferation and metastasis of NSCLC cells (146). Circ-0109320 is significantly more expressed in NSCLC and associated with more advanced staging and lymph node metastasis of NSCLC *via* sponging of miR-595 and subsequently upregulates E2F7 expression (147). A novel circRNA (circHIPK3) increases STAT3 expression by inhibiting miR-124-3p in STK11 mutant lung cancer cells (148). Moreover, silencing circHIPK3 results in the reduction of cell proliferation, migration invasion, and promotion of macroautophagy/autophagy *via* MIR124-3p-STAT3-PRKAA/AMPKα signaling in STK11 mutant lung cancer (148). Overexpression of circAKT3 significantly promotes proliferation, migration, and invasion by sponging miR-330-5p, resulting in increased HMGB3 expression in non-small-cell lung cancer cells (149). circRNA-010763 promotes the growth and invasion of lung cancer through the regulation of the circRNA-010763/miR-715/c-Myc axis (150). Circ-THBS1 is highly expressed in patients with metastatic NSCLC and promotes the cell proliferation of LUAC cells by sponging miR-129-5p and SOX4 overexpression (151). There is also evidence that circ- 0012673 facilitates lung cancer cell proliferation and invasion *via* the miR-320a/LIMK18521axis (152). Several other regulatory cascades comprising circRNA, miRNA, and mRNA have been reported in lung cancer, including circ-MAN2B2/miR-1275/FOXK1 (153), circ- 103809/miR-4302/ZNF121/MYC (154), circ-0020123/miR-144/ZEB1 and EZH2 (155), circ-FADS2/miR-498 (156), circ-0026134/miR-1256/miR-1287 (157), circ -0016760/miR-577/ZBTB7A (158), circ-0016760/miR-1287/GAGE1 (159), circ-BANP/miR-503/LARP (160), circ-001569/Wnt/β-catenin (161), circ -FOXM1/miR-1304-5p/PPDPF/MACC1 (162), Circ-FGFR3/miR-22-3p/Gal-1,p-AKT, p-ERK1/2 (163), circ-PVT1/miR-125b/E2F2 (164), circ-PVT1/miR-497/Bcl-2 (165), circ-0000735/miR-1179/miR-1182 (166), circ-100146/miR-361-3p/SF3B3 (167), circ-0043278/miR-520f/ROCK1, CDKN1B and AKT3 (168), circ-RAD23B/miR-593-3p/CCND2 and circ -RAD23B/miR-653-5p/TIAM1 (169), circ-0003645/miR-1179/TMEM14A (170), circ-P4HB/miR-133a-5p/vimentin (171), circ-0046264/miR-1245/BRCA2 (172), and circ- 100395/miR-1228/TCF21 (54).

Extensive evidence has demonstrated that circRNAs play a significant regulatory role in cell cycle transition. The dysregulation expression of circRNAs leads to tumor cell cycle progression.

circRNAs are involved in the regulation of G1/S checkpoint and participate in the development and progression of lung cancer. p21 is a cyclin-dependent kinase inhibitor that binds and inhibits the catalytic activity of Cdk2, leading to G1-phase cell cycle arrest (173). circ-Foxo3 binds to CDK2 and p21, giving origin to a circ-Foxo3-p21-CDK2 ternary complex that inhibits CDK2-dependent G1/S cell cycle progression (96). In addition, circ-0013958 acts as a sponge of miR-134, with subsequent upregulation of oncogenic cyclin D1 that plays a pivotal in the development of non-small-cell lung cancer (174). Circ-0006916 inhibits miR-522, promoting the G0/G1 progression of NSCLC (175). Circ-TP63 is highly expressed in LUSC tissues and correlates with a more advanced TNM stage. circ-TP63 competes for binding with miR-873-3p and then upregulates FOXM that, in turn, upregulates CENPA and CENP, thus promoting cell cycle progression (176).

### circRNAs regulate the invasion and metastasis of lung cancer cells

Mounting evidence revealed that the aberrant expression of circRNAs is implicated in the invasion and metastasis of lung cancer —for example, circPTK2 acts a sponge for miR-429/miR-200b-3p, thus promoting TGF-β-induced EMT *via* TIF1γ and the invasiveness of NSCLC cell (15).

Circ-ABCB10 is also increased in NSCLC cell lines, and the knockdown of circ-ABCB10 suppresses NSCLC cell migration by promoting microRNA miR-1252 expression and suppressing Forkhead box 2 (FOXR2) (177). Circ-MAN2B2 promotes lung cancer cell invasion *via* the miR-1275/FOXK1 axis (153). The upregulated expression of circ-0067934 is associated with the invasiveness and migration of tumor cells and the overexpression of some EMT-associated markers such as N-cadherin (178). Circular RNA cESRP1 acts as a sponge for miR-93-5p and targets Smad 7/p21(CDKN1A), causing the inhibition of TGF-β-mediated EMT progress in lung cancer (179).

Circ-BANP promotes the migration, invasiveness, and increased expression of LARP1 in lung cancer cells *via* miR-93-5p and the inhibition of miR-503, respectively (160).

CircAGFG1 is elevated in NSCLC tissues and promotes invasion, migration, and epithelial–mesenchymal transition *via* the circ-AGFG1/miR-203/ZNF281 axis (180).

Circ-PTPRA suppresses EMT in NSCLC cell lines through the circ-PTPRA/miR-96-5p/RASSF8/E-cadherin axis and is downregulated in NSCLC tumor (135). Naringenin inhibits cell migration and invasion by regulating the circ-FOXM1/miR-3619-5p/SPAG5 axis in lung cancer (181). Several other regulatory cascades including circRNA, miRNA, and mRNA have been reported in lung cancer: circ-SATB2/miR-326/FSCN1 (124), circ-BIRC6/miR-145 (130), circ-NT5E/miR-134 (131), circ-ARHGAP10/miR-150-5p/GLUT1 (132), circ-FOXM1/miR-338-3p/RAB14 (134), circ-PTPRA/miR-96-5p/RASSF8 (135), circ-0102231/miR-145/RBBP4 (136), circ -100565/miR-506-3p/HMGA2 (138), circ-0001320/miR-558/TNFAIP1/TPM1 (126), circ-001010/miR-5112/CDK4 (126), circ-0087862/miR-1253/RAB3D (140), circ-CRIM1/miR-93 and miR-182 (141), circ-11780/miR-544a/FBXW7 (145), circ -0072088/miR-377-5p/NOVA2 (146), circ-0109320/miR-595/E2F7 (147), circ-HIPK3/miR124-3p-STAT3 (148), circ-AKT3/miR-330-5p/HMGB3 (149), circ- 010763miR-715/c-Myc (150), and circ-0012673/miR-320a/LIMK18521 axis (152).

### circRNAs regulate the stemness and resistance of lung cancer stem cells

NSCLC is often a stubborn disease characterized by chemoresistance that represents a significant clinical challenge and contributes largely to disease progression, recurrence, and mortality (182). Despite the recent therapeutic advances (i.e., targeted therapy and immunotherapy), chemotherapy is still the backbone treatment for patients with advanced NSCLC cancer. Either cisplatin or carboplatin is used in combination with gemcitabine, vinorelbine, pemetrexed, or taxanes (docetaxel or paclitaxel) (183), while most patients with NSCLC are sensitive to chemotherapy at the early and the late stage. They show drug resistance that requires the identification of novel targets and development of more personalized medicine in the future (184). Despite intense efforts to overcome such resistance in lung cancer and other cancer types using novel agents (alone or in combination with chemo- and radiotherapy), the underlying mechanisms conferring this resistant phenotype in lung cancer remain largely unknown (185). It is now well established that CSCs constitute a unique subset of cells which are distinct from the bulk of tumor cells by their exclusive ability to perpetuate the growth of a malignant population of cells. This may explain the ineffectiveness of many conventional therapies and patient relapse (186). The ability of CSC to self-renew and differentiate results in tumor growth, progression, metastasis, and drug cancer treatment failure (187). This multi-drug resistance is mostly due to channel proteins that expel anticancer drugs, leading to decreased drug cell concentration (187). The stemness markers of lung cancer stem cells mainly include ALDH1, ABCG2, CD44, CD133, NANOG, and SRY-box transcription factor 2 (SOX2) (188). However, a recent study showed that circRNAs underpin cancer cell stemness by the upregulation of these stemness markers (189).On the contrary, circ-SOX4 suppresses cell proliferation, self-renewal, migration, and the invasiveness of lung tumor-initiating cells. Additionally, circ-SOX4 activates the Wnt/β-catenin pathway to maintain the stemness of lung cancer stem cells (190). Depletion of circPOLA that acts as a sponge for miR-326 with the subsequent upregulation of the G protein subunit beta 1 (GNB1) expression (56) leads to reduction of sphere formation ability, ALDH1 activity, and stemness marker expression of lung cancer cells. Consistently, the high expression of circPOLA in lung cancer tissues is associated with poor prognosis. CDR1as is upregulated in CDDP-resistant NSCLC cells, whereas its overexpression enhances the stemness signatures (SOX2, OCT4, and Nanog) *via* the miR-641/HOXA9 axis and confers resistance to CDDP-sensitive NSCLC cells (191).

Circ-PVT1 is highly expressed in LUAC cell lines and tissues and related to CDDP and MTA. Circ PVT1 mediates CDDP and MTA resistance *via* the miR-145-5p/ABCC1 axis, and Circ PVT1 knockout sensitizes tumor cells to CDDP and MTA (192). Mao et al. reported that CDR1-AS is also upregulated in cell lines and LUAC tissues and cell lines and confers resistance of patients to paclitaxel (PTX) and CDDP *via* the epidermal growth factor receptor (EGFR)/phosphatidylinositol 3-kinase (PI3K) signaling pathway (193).

cESRP1 is significantly downregulated in the chemoresistant cells and augments drug sensitivity by sponging miR-93-5p in SCLC, thereby upregulating CDKN1A, and subsequently inhibits transforming growth factor-β-mediated epithelial–mesenchymal transition *via* miR-93-5p (179). Circ-0085131 acts as a ceRNA of miR-654-5p and upregulates the expression of autophagy-associated factor ATG7, thereby modulating cell chemoresistance. Circ-0085131 is more expressed in NSCLC tumor tissue than in the adjacent normal tissue, and the higher expression is associated with the recurrence and poorer survival of NSCLC (194). He and colleagues have shown that circ-0000567 and circ-0006867 are upregulated and downregulated, respectively, in two gefitinib-resistant cell lines. The Gene Ontology and Kyoto Encyclopedia of Genes and Genomes pathway analysis indicates that dysregulation of circRNAs might play an important role in the development of acquired resistance to gefitinib in NSCLC (195). Circ AKT3 inhibits the cisplatin sensitivity of lung cancer cells *via* the miR-516b-5p/STAT3 axis-mediated glycolysis balance (196). Circ-AKT3 is upregulated in lung cancer tissues and cells, and knockdown of circAKT3 improves the cell sensitivity to CDDP and reduces glycolysis. Consistently, the inhibition of HIF-1α-dependent glycolysis attenuates the circAKT3-induced increase of chemo-resistance in A549 cells. Circ-0001946 promotes the viability, proliferation, migration, and invasion of NSCLC cells and inhibits cell apoptosis. Circ-0001946 has also been proven to reduce the sensitivity of NSCLC cells to CDDP by regulating the nucleotide excision repair (NER) signaling pathway (197). Circ-0076305 is elevated in CDDP-resistant NSCLC tissues and cells and exerts this effect *via* targeting miR-296-5p and enhancing the expression of STAT3 (198). Circ 0004015 enhances the resistance of HCC827 to gefitinib by sponging miR-1183 and upregulating the expression of PDPK1 (199). Circ-0003998 targets miR-326 and inhibits the miR-326-mediated effect on chemosensitivity (200). It is highly expressed in LUAC tissues and docetaxel-resistant cell lines, and its depletion decreases chemoresistance, inhibits proliferation, and enhances apoptosis in docetaxel-resistant LUAC cells.

Circ MTDH.4 regulates AEG-1 expression *via* miR-630 and promotes chemoresistance and radio-resistance in NSCLC cells (201). Circ-ABCB10 increases the expression of AK4, promotes lung cancer progression, and sensitizes lung cancer cells to cisplatin *via* sponging miR-556-3p (202). Knockdown of circ-103762 promotes CHOP expression and inhibits multidrug resistance in NSCLC (203). Wang et al. have shown that circRNA 002178 enhances PDL1 expression and induces T cell exhaustion *via* targeting miR-34. Furthermore, circ-002178 is detectable in the exosomes of plasma from LUAC patients and is a potential biomarker for the early diagnosis of LUAC (125). Circ-FGFR1 enhances the expression of the motif chemokine receptor 4 (CXCR4) *via* miR-381-3p, thus promoting NSCLC progression and resistance to anti-programmed cell death 1 (PD-1)-based therapy (204).

Depletion of circ-0007385 suppresses cell proliferation, migration, and invasion in NSCLC cells and cisplatin (DDP) resistance. Circ-0007385 is highly expressed in NSCLC tissues and cell lines and associated with poor overall survival.

Circ-0007385 regulates HMGB1 expression and promotes the chemoresistance in NSCLC cells *via* sponging miR-519d-3p (205).

Circ-0002483 is downregulated in NSCLC cells, enhances the sensitivity of NSCLC cells by sponging miR-182-5p, and regulates its target gene growth factor receptor-bound protein2 (Grb2), forkhead box protein O1 (Foxo1), and forkhead box protein O3 (Foxo3) (57). One group investigated the expression profile of circRNAs involved in the development of early-stage lung adenocarcinoma and found that circRNA 404833 and circ 406483 might be exceptional potential candidates as early diagnostic biomarkers for lung cancer. Among them, circ-404833, through targeting miR-149-5p, regulates the cell motility and gefitinib resistance in lung adenocarcinoma (172). It has been shown that Circ-SMARCA5 is downregulated in non-small-cell lung cancer. Its low expression was negatively correlated with tumor size, lymph node metastasis, and TNM stage but positively correlated with disease-free survival and overall survival (OS). Over-expression inhibits the cell proliferation of NSCLC and enhances the chemosensitivity to cisplatin and gemcitabine (206). Overall, these studies illustrate that circRNAs have significant regulatory functions in the chemotherapy resistance of lung cancer.

### circRNAs regulate the tumor metabolism and tumor microenvironment of lung cancer

Tumor metabolic reprogramming is a hallmark of cancer. In terms of energy metabolism, the glycolysis pathway often has abnormal activation in cancer cells (207). There is an increasing number of evidence that have been uncovered which show that circRNAs, through a different mechanism, can activate the glycolysis pathway and participate in the progression of cancer. One such study has shown that circ SLC25A16 can accelerate the glycolysis and proliferation of NSCLC cells. Mechanistic research demonstrated that circ-SLC25A16 can act as a sponge for miR-488-3p and elevate the expression of HIF-1α that is a target gene of miR-488-3p. In turn, HIF-1α activates lactate dehydrogenase A (LDHA) by facilitating its transcription (208). It was suggested that the overexpression of Circ-CRIM1 inhibited LUAC cell migration, invasion, EMT, glycolysis, and tumor growth through reducing the expression of miR-125b-5p and resulted in the enhance expression of BTG2 (142). The depletion of circ-ACACA inhibited the proliferation and migration of NSCLC cells and also reduced the glycolysis rate. The details of this molecular mechanism include circ-ACACA by its sponging of miR-1183 and inactivating the PI3K/PKB signaling pathway (209). Circ-ENO1, which is derived from its host gene ENO1, was upregulated in LUAC. The overexpression of circ-ENO1 can promote glycolysis, proliferation, migration, and EMT and induce apoptosis in LUAC cells. circ-ENO1 acted as a ceRNA to interact with miR-22-3p, and an upregulated ENO1 expression promoted glycolysis and tumor progression in LUAC (58). Moreover, it was reported that Circ-CRIM1, through targeting miR-125b-5p, results in an increase of the expression of BTG2, leading to the promotion of glycolysis in lung adenocarcinoma cells (142). Circ-NFIX was highly expressed in NSCLC. The overexpression of circNFIX can promote NSCLC cell viability, migration, invasion, and glycolysis *in vitro* and hampered tumor growth *in vivo*. Mechanistic analyses have demonstrated that circ-NFIX acted as a molecular sponge of miR-212-3p and upregulated the expression of ADAM10 (210).

TME is the product of the crosstalk between different cell types and plays a crucial role in the progression, metastasis, and therapeutic treatment of cancer (211). Tumor immune escape refers to the phenomenon of tumor cells growing and metastasizing *via* various mechanisms to avoid recognition and attack by the immune system. The mechanism of tumor immune escape includes immunosuppression. Programmed death 1/programmed death-ligand 1 (PD-1/PD-L1), known as an immune checkpoint, is an important component of tumor immunosuppression (212). It has also been reported that circ-002178, from the exosomes of plasma from LUAC patients, was highly expressed in LUAC tissues and LUAC cancer cells. Functional studies have demonstrated that circ-002178 could enhance PDL1 expression *via* sponging miR-34 in cancer cells to induce T cell exhaustion (125). Circ-CPA4 was recently identified to be upregulated in NSCLC cells and cancer tissues. Compared to human bronchial epithelial cells and their paired clinical normal adjacent tissues, circ-CPA4 regulated cell growth, mobility, stemness, and drug resistance in NSCLC cells and inactivated CD8+ T cells in the tumor immune microenvironment through the let-7 miRNA/PD-L1 axis (59). Interestingly, a recent study showed that circ0000284 facilitated the progression of NSCLC by upregulating the PD-L1 expression as a competing endogenous RNA (ceRNA) of miR-377 (213). Therefore, regulating the PD-1/PD-L1 expression by targeting related circRNAs may be a direction of future immune checkpoint therapeutic research. Furthermore, some circRNAs regulate the cytokine expression to influence the function of immune cells—for example, ciRS-7 interacts with miR-7 to modulate the expression of NF-κB, modulating the activities of immune cells and affecting the development of lung cancer (214, 215). The non-cellular components of TME mainly include the extracellular matrix (ECM) and hypoxia environment. ECM, composed of matricellular proteins, elastin, collagen, and cytokines, provides the structural and functional bases for tumor cellular function (216). Circ-0000064 was reported to promote cell proliferation and inhibit apoptosis by the regulation of MMP-2 and MMP-9, participating in the destruction of the histological barrier as well as the invasion of cancer cells (217). The metabolism of cellular components in TME may result in a hypoxia environment, causing the generation of hypoxia-inducible factors (HIFs) to modulate the progression of tumor (218). A previous study has shown that upregulated circPIP5K1A inhibits miR-600 to enhance the HIF-1α expression to promote the migration and proliferation of NSCLC cells (219). Furthermore, circ-FAM120A expression was found to be downregulated in hypoxic LUAC, while HIF-1α expression increased at the same time, both promoting tumor growth (220). Therefore, circRNAs are identified to influence the hypoxia environment of TME in lung cancer.

## The diagnostic and prognostic value of circRNAs

The high stability, abundance, and spatiotemporal-specific expression of circRNAs make them ideal biomarkers for liquid biopsy. The important functions of circRNAs in blood cells suggest that the dysregulation of circRNA expression in blood cells is likely to contribute to the occurrence and progression of lung cancers—for instance, in NSCLC patients, Tan et al. found that F-circEA, an f-circRNA originating from the EML4-ALK fusion gene, was exclusively expressed in the plasma of patients with the EML4-ALK fusion. Therefore, plasma F-circEA may serve as a liquid biopsy biomarker to diagnose NSCLC patients with EML4-ALK translocation and guide the targeted therapy for NSCLC patients in this subgroup (129). In a recent study, Luo et al. measured the expression levels of two circRNAs in the plasma samples of 231 lung cancer patients and 41 healthy controls using reverse transcription droplet digital PCR (221). They identified has circ-0000190 as a circRNA biomarker in human blood plasma that can predict the survival outcomes of lung cancer patients (221). Furthermore, the increased expression of circ-0000190 in plasma was also correlated with poor response to systemic therapy and immunotherapy in lung cancer patients (221). Similarly, Li et al. observed that SCLC patients with lower exosomal circFLI1 expression levels experienced longer disease remissions, indicating its prognostic power in SCLC. The authors also suggested that serum exosomal circFLI1 may be used as a biomarker that can monitor the clinical 191response to chemotherapy in SCLC patients (222). Notably, they observed that SCLC patients with lower exosomal circ-FLI1 expression levels experienced longer disease remissions, indicating its prognostic power in SCLC. The authors also suggested that serum exosomal circFLI1 may be used as a biomarker that can monitor the clinical response to chemotherapy in SCLC patients (222).

The main evaluative criterion of the diagnostic value of circRNAs is AUC in the ROC analysis. Previous studies have shown that many circRNAs may serve as potential diagnostic biomarkers. Circ 0000729 had an area under the receiver operating characteristic curve (AUC-ROC) of 0.815 for discriminating LUAC from normal controls (223). Circ-0000064, being located in the cytoplasm circRNA, was reported to be upregulated in lung cancer tissues and lung cancer cell lines. Its aberrant expression was correlated with several clinical characteristics, including T stage, lymphatic metastasis, and TNM stage, which represents a novel potential biomarker for lung cancer diagnosis (217). It was suggested that circ-102231 was highly expressed in lung adenocarcinoma tissues and associated with advanced TNM stage, lymph node metastasis, and poor overall survival of lung cancer patients. The depletion of circ-102231 significantly restrains lung cancer cell proliferation and its invasion ability. It counts as a potential diagnostic biomarker for lung cancer patients. Its area under the ROC curve was 0.897, and circ 102231 showed good sensitivity and specificity of 81.2 and 88.7%, respectively (224). Circ-0014130 was elevated in NSCLC tissues. Its high expression correlated with TNM stage and lymphatic metastasis. The area under the receiver operating characteristic curve of circ-0014130 in NSCLC was 0.878, which showed good diagnostic potential. Furthermore, *via* gene oncology analysis and pathway analysis, it was indicated that circ-0014130 could participate in NSCLC development and could be used as a potential NSCLC diagnostic biomarker (225). Li et al. reported that the circ-0075930 expression levels were significantly higher in NSCLC cell lines and tissues. Its high expression was correlated with tumor size and lymph node metastasis. circ-0075930 had an AUC-ROC of 0.756 for discriminating NSCLC from normal controls, with sensitivity and specificity of 76.2 and 72.1%, respectively (226). Lu et al. reported that the circ0001715 levels were significantly higher in lung adenocarcinoma patients versus healthy controls. Its high expression significantly correlated with TNM stage and distant metastasis. Furthermore, the ROC curve analysis showed that the AUC of circ-0001715 was 0.871. The univariate and multivariate survival analyses showed that the plasma circ 0001715 level was an independent prognostic factor for the OS (227). The ROC curve indicated that the AUC of circ FOXO3 was 0.782, and the sensitivity and specificity of diagnosing NSLCL with circRNA-FOXO3 reached 80.0 and 73.3%, respectively (228).

circRNAs have stable circular structures which make them more stable and particularly attractive as liquid biopsy biomarkers. Circ-0013958 was elevated in lung adenocarcinoma tissues, cells, and plasma. Its high expression was associated with TNM stage and lymphatic metastasis. The area under the receiver operating characteristic curve was 0.815. In addition, circ-0013958 could be a sponge of miR-134 and thus upregulated oncogenic cyclin D1. It may be a potential non-invasive biomarker for the early detection and screening of lung adenocarcinoma (174). Circ-FARSA, derived from exon 5–7 of the FARSA gene, was elevated in cancerous tissues and patients’ plasma. The elevated expression of circ-FARSA significantly promoted cell migration and invasion in NSCLC cells. Circ-FARSA could inhibit the activity of miR-330-5p and miR-326, thereby relieving their inhibitory effects on oncogene fatty acid synthase. A further analysis of the diagnostic value of plasma circ-FARSA in distinguishing NSCLC patients from non-cancer patients indicated the area under the ROC curve to be 0.71. This evidence indicated that plasma circFARSA could be a noninvasive biomarker for this NSCLC malignancy (229).

Circ-0067934 was shown to be markedly overexpressed in NSCLC tissues and cell lines. Its high expression was correlated with TNM stage, lymph node status, and distant metastasis in NSCLC patients. A higher expression of circ-0067934 results in significantly poorer survival, and the results of a multivariate Cox proportional hazard analysis indicated that circ-0067934 was an independent poor prognostic factor for patients with NSCLC. The depletion of circ-0067934 significantly suppressed NSCLC cell proliferation, migration, and invasion (178). Circ 100876 has been reported to be significantly elevated in NSCLC tissues than their adjacent nontumorous tissues. Its high expression correlated with lymph node metastasis, tumor stage, and poor overall survival in NSCLC. It might serve as a potential prognostic biomarker for NSCLC (230). It was suggested that circ-FOXM1 is highly expressed in NSCLC tissues and closely correlated with lymph node invasion, higher TNM stage, and unfavorable prognosis (162). The depletion of circ FOXM1 significantly restrains the growth, migration, and invasion of NSCLC cells. A further study has shown that Circ FOXM1 upregulated the expression of PPDPF and MACC1 *via* inhibiting the activity of miR-1304-5p (162). The above-mentioned examples demonstrated that some circRNAs could be promising biomarkers for the diagnosis and prognosis of lung cancer.

Lin et al. have shown that circ PRKCI was overexpressed in lung adenocarcinoma tissues and promoted the proliferation and tumorigenesis of lung adenocarcinoma. This group established a nude mouse xenograft model. Compared with the siRNA-transfected cell-derived tumors, the si-circPRKCI-transfected cell-derived tumors were smaller and lighter in weight (231). The therapeutic potential of circPRKCI was evaluated *via* an intratumoral injection of cholesterol-conjugated si-circPRKCI and control siRNA in patient-derived tumor xenografts (PDTX). The results indicated that si-circPRKCI significantly inhibited the growth of PDTX *in vivo*, suggesting that circPRKCI may be a promising therapeutic target for LUAC (231).

## Conclusion and future direction

With the rapid development of high-throughput sequencing technologies and bioinformatics approaches, increasing evidence have uncovered that circRNAs play an important role in several human physiological and pathological processes, such as adipogenesis (43), lung inflammation (232), brain development (233), repair of ischemic muscle injury (234), mitochondrial metabolism (46), and cancer progression (235). Lung cancer is the leading form of cancer in terms of both morbidity and mortality. Due to the lack of effective diagnostic markers, many lung cancer patients are approaching the advanced stage. Recently, with the widespread use of computed tomography and magnetic resonance imaging, incidental renal masses are increasingly being detected. However, there is an urgent need for specific biomarkers to allow for the early identification of postoperative lung cancer recurrence and metastasis. Screening and early diagnosis of lung cancer are critical for improving the treatment efficacy and reducing the mortality of patients with lung cancer and have been identified as a research priority. As a novel noncoding RNA, circRNA influences the initiation and progression of lung cancer through participation in diverse processes of lung cancer pathogenesis, including proliferation, migration, invasion, apoptosis, cell cycle, stemness of lung cancer stem cell, tumor metabolism of lung cancer, radiotherapy, and chemotherapy resistance. All of these findings suggest that circRNAs play pivotal roles in the pathological progression of cancer and may be useful as cancer biomarkers. circRNAs can be easily detected from a wide range of biological samples due to their high stability and specificity, representing as ideal diagnostic and prognostic biomarkers in lung cancer.

Previously identified lung CSC subsets have been shown to confer resistance to conventional chemotherapeutics, biological molecules, targeted therapies, and radiotherapy used in the current management of lung cancer. Specific targeting of cancer stem cells in combination with first-line chemotherapeutic agents holds great promise as a strategy to overcome chemoresistance, tumor relapse, and metastasis. A number of CSC markers have been identified and studied. These include ALDH1, ABCG2, CD44, CD133, NANOG, and SRY-box transcription factor 2 (SOX2), all of which have been linked to chemoresistance in a number of first line anti-cancer therapies—for example, axitinib is an oral, potent, small molecule ATP-competitive multitarget tyrosine kinase inhibitor. It inhibits the CSC marker ABCG2. Axitinib has also been shown to reverse multidrug resistance *via* ABCG2 inhibition both *in vitro* and *in vivo*. The axitinib–doxorubicin combination treatment promoted the intracellular accumulation of doxorubicin within the side population of CSC cells and significantly enhanced the cytotoxic effects of doxorubicin (236). In addition, a recent finding by our group was that, under normoxic conditions, YTHDF1 is highly expressed in non-small-cell lung cancer cancerous tissues and cell lines to promote cell proliferation *via* increasing cell-cycle-related factor expression. However, under hypoxic conditions or stressful chemotherapy conditions, YTHDF1 is downregulated, which leads to reduced Keap1 mRNA translational efficiency and Nrf2 protein stabilization (5).

With the deepening research on circRNAs, it is very important to choose appropriate and efficient database and analysis software for the precise analysis of circRNAs. We will also summarize recently developed database that have been developed to provide tremendous valuable information. CircBase mainly collect circRNA information of multiple species, including human, mouse, Caenorhabditis elegans, D. melanogaster, Latimeria chalumnae, and coelacanth (237). DeepBase, a database that integrate all public deep-sequencing data, provides an integrative evaluation of miRBase-annotated miRNA genes and other known ncRNAs and explores the expression patterns of miRNAs and other ncRNAs (238). CircNet database provides the following information related to circRNAs: novel circRNAs, integrated miRNA–target networks, expression profiles of circRNA isoforms, genomic annotations of circRNA isoforms, and sequences of circRNA isoforms. In addition, the CircNet database that provides tissue-specific circRNA expression profiles and circRNA–miRNAgene regulatory networks illustrates the regulation between circRNAs, miRNAs, and genes (239). circRNADb contains human circular RNAs with protein-coding annotations. The detailed information of the circRNA included genomic information, exon splicing, genome sequence, IRES, open reading frame (240). CircInteractome database is used for mapping RBP- and miRNA-binding sites on human circRNAs. circInteractome also carried out the following functions: identify potential circRNAs which can act as RBP sponges, design junction-spanning primers for the specific detection of circRNAs of interest, design siRNAs for circRNA silencing, and identify potential IRES (241). CSCD is a database for cancer-specific circular RNAs. It includes the following information: the microRNA response element sites and RNA-binding protein sites for each circRNA, the predicted potential open reading frames of circRNAs, and the splicing events in the linear transcripts of each circRNA (242). CIRCpedia v2 is a database for comprehensive circRNAs with expression features in various cell types/tissues, including disease samples. It also can perform a conservation analysis of circRNAs between humans and mice (243). TSCD is for Tissue-Specific CircRNA Database, containing information about tissue-specific circRNAs in human and mouse (244). ExoRBase is a database of circRNA, lncRNA, and mRNA derived from RNA-seq data analyses of human blood exosomes (245). circRNA Disease is a database that provides a user-friendly interface for searching disease-associated circRNAs (246). CircVAR is a database used to collect SNPs and small insertions and deletions in putative circRNA regions and to identify their potential phenotypic information (247). TransCirc is a specialized database that provides comprehensive evidences supporting the translation potential of circular RNAs (248). Circ2GO is a database that contains information on circRNAs and their function, processes, and miRNA targets (249). Although the listed databases have different advantages, there are still many problems, such as prediction overlap, lack of a unified identification standard, the results predicted by different databases that vary greatly, and the lack of a uniform identification standard. Therefore, when using these informatics methods, it is necessary to set specific conditions and improve the certainty of the data.

Exosomes can mediate cell-to-cell communication in both physiologic and pathologic processes. Exosomes contain RNAs, proteins, and lipids, all of which affect exosome functions and reflect cell characteristics. The major nucleic acids of exosomes are RNAs that contain microRNA (miRNA), tRNA, and long noncoding RNA (lncRNA) as well as circRNAs (250). Exosomes can serve as a novel cellular communication bond by transferring their contents to target cells in a lung cancer microenvironment, thereby regulating lung cancer cell progression (251)—for example, under hypoxic conditions, miR-23a was significantly increased in exosomes from lung cancer cells, and exosomes transferred miR-23a to endothelial cells. miR-23a directly inhibited its target prolyl hydroxylase 1 and 2 (PHD1 and 2) expression, resulting in the accumulation of hypoxia-inducible factor-1α in endothelial cells. Consequently, exosomes derived from hypoxic lung cancer cells increased endothelial permeability and cancer cell transendothelial migration *in vitro* and enhanced neovascularization and tumor growth *in vivo* (252). In addition, tumor-derived exosomes carry immunosuppressive molecules, transfer these cargos to immune cells, and directly or indirectly suppress the functions of immune cells, thereby promoting tumor progression (253). circRNAs can be packaged into exosomes and transferred to receptor cells, further impacting the development of diseases. A recent study by Zhang N et al. reported that circSATB2 was highly expressed in NSCLC cancer exosomes, which can promote the proliferation, migration, and invasion of NLCSC cells. Exosomes which contained circ SATB2 can be taken up by NLCSC cells to participate in cell–cell communication, further affecting the progression of NSCLC cells and the proliferation of normal bronchial epithelial cells. Moreover, exosomal circSATB2 expression was related to lung cancer lymphatic metastasis. These results indicate that exosomal circSATB2 has the potential to act as a blood detection index for the diagnosis of lung cancer and lung cancer metastasis with high sensitivity and specificity (124). Similarly, it has also been reported that circRNA 002178 from the exosomes of plasma from LUAC patients was highly expressed in LUAC tissues and LUAC cancer cells. Functional studies have demonstrated that circRNA-002178 could enhance PDL1 expression *via* sponging miR-34 in cancer cells to induce T cell exhaustion. Interestingly, the area under the curve (AUCs) of circRNA-002178 was 0.9956, which was higher in the exosomes derived from cancer cells than the exosomes derived from normal human bronchial epithelial cells, and could serve as a novel diagnosis biomarker for lung cancer (125).

TME is the product of the crosstalk between different cell types and plays a crucial role in the progression, metastasis, and therapeutic treatment of cancer (211). The TME in both primary and secondary lung tumors is recognized as a target-rich environment for the development of novel anticancer agents. It has been reported that circRNA could modulate immune responses in the tumor microenvironment of lung cancer and be involved in lung cancer progression—for instance, ciRS-7 interacts with miR-7 to modulate the expression of NF-κB, modulating the activities of immune cells and affecting the development of lung cancer (214, 215). Mechanistic insights into the tumor-reprogrammed microenvironmental landscape in lung cancer together with the development of drugs that specifically inhibit the components of the landscape have ushered in a new era of cancer medicine.

Although there are many studies that have reported the biological function of RNA in lung cancer, there are still many problems surrounding circRNAs that remain to be clarified. First, the mechanism of expression and localization of circRNA is still unclear. Second, many new technologies, such as antisense oligonucleotide and CRISPR/Cas9-mediated knockout could be used to interfere the endogenous function of circRNA that promote cancer progression. Exosomes can transfer biologically active molecules between lung cancer cells and their microenvironment, but the mechanisms of exosome-related circRNA involved in lung cancer progression need to be illuminated. Finally, as circRNAs are stable and have unique structural conformations, additional investigations are still required to decipher the complex molecular mechanism so that circRNA could be implemented clinically through translational medicine, especially in the case of a disease such as lung cancer.

## Author contributions

WR and YY contributed to the direction and guidance of this review and prepared the figure. JP, LM and XJ drafted the manuscript and revised the manuscript; All authors read and approved the final manuscript.

## Funding

This work was supported by Yunnan Fundamental Research Projects (grant no. 202101AS070043), Kunming Science and Technology Plan Project (grant no. 2020-1-H-003), Kunming Medical University Graduate Student Innovation Fund (grant no. 2022B24), and The First People’s Hospital of Yunnan Province Clinical Medicine Center Opening Project (grant no. 2022LCZXKF-XZ02).

## Conflict of interest

The authors declare that the research was conducted in the absence of any commercial or financial relationships that could be construed as a potential conflict of interest.

## Publisher’s note

All claims expressed in this article are solely those of the authors and do not necessarily represent those of their affiliated organizations, or those of the publisher, the editors and the reviewers. Any product that may be evaluated in this article, or claim that may be made by its manufacturer, is not guaranteed or endorsed by the publisher.

## Abbreviations

LUAC, lung adenocarcinoma; NSCLC, non-small-cell lung cancer; LUSC, lung squamous cell carcinoma; LC, lung cancer; SCLC, small-cell lung cancer; pre-mRNA, premessenger RNA; m6A, N6-methyladinosinek; ORF, open reading frame; hnRNPs, heterogeneous nuclear ribonucleoproteins; DHX9, ATP-dependent RNA helicase A; AGO2, argonaute 2; RNAi, RNA interference; circRNAs, circular RNAs; ncRNAs, non-coding RNAs; miRNAs, microRNAs; ecircRNAs, exonic circRNAs; ElciRNAs, exon–intron circRNAs; ciRNAs, intronic circRNAs; Pol II, RNA polymerase II; snRNP, small nuclear ribonucleoprotein; RBPs, RNA-binding proteins; ceRNA, competing endogenous RNA; EMT, epithelial–mesenchymal transition; TNM, tumor–node–metastasis; OS, overall survival; ROC, receiver operating characteristic; ASOs, anti-sense oligonucleotides; CRISPR/Cas9, clustered regularly interspaced short palindromic; repeats-associated nuclease Cas9; circ-AS, circRNA alternative splicing; ICSs, flanking intronic complementary sequences; CSC, cancer stem cell.
